# Glioblastoma chemoresistance: roles of the mitochondrial melatonergic pathway

**DOI:** 10.20517/cdr.2020.17

**Published:** 2020-06-16

**Authors:** George Anderson

**Affiliations:** CRC Scotland & London, Eccleston Square, London SW1V 1PG, UK.

**Keywords:** Glioblastoma, glioblastoma stem-like cells, melatonin, *N*-acetylserotonin, mitochondria, metabolism, circadian, treatment, chemoresistance, tyrosine receptor kinase B

## Abstract

Treatment-resistance is common in glioblastoma (GBM) and the glioblastoma stem-like cells (GSC) from which they arise. Current treatment options are generally regarded as very poor and this arises from a poor conceptualization of the biological underpinnings of GBM/GSC and of the plasticity that these cells are capable of utilizing in response to different treatments. A number of studies indicate melatonin to have utility in the management of GBM/GSC, both *per se* and when adjunctive to chemotherapy. Recent work shows melatonin to be produced in mitochondria, with the mitochondrial melatonergic pathway proposed to be a crucial factor in driving the wide array of changes in intra- and inter-cellular processes, as well as receptors that can be evident in the cells of the GBM/GSC microenvironment. Variations in the enzymatic conversion of *N*-acetylserotonin (NAS) to melatonin may be especially important in GSC, as NAS can activate the tyrosine receptor kinase B to increase GSC survival and proliferation. Consequently, variations in the NAS/melatonin ratio may have contrasting effects on GBM/GSC survival. It is proposed that mitochondrial communication across cell types in the tumour microenvironment is strongly driven by the need to carefully control the mitochondrial melatonergic pathways across cell types, with a number of intra- and inter-cellular processes occurring as a consequence of the need to carefully regulate the NAS/melatonin ratio. This better integrates previously disparate data on GBM/GSC as well as providing clear future research and treatment options.

## Introduction

Treatment-resistance is not uncommon in patients with glioblastoma (GBM), especially in recurrent GBM^[1]^. This arises from the complexity of the wide array of diverse intracellular and extracellular processes that underpin GBM and the glioblastoma stem-like cells (GSC) from which they arise^[2]^. A plethora of pathophysiological processes are associated with GBM/GSC including microRNAs (miRNAs), AMP-activated protein kinase (AMPK), 14-3-3 proteins, mammalian target of rapamycin (mTOR), aryl hydrocarbon receptor (AhR), sirtuins, histone deacetylation (HDAC), endoplasmic reticulum (ER), sphingosine-1-phosphate (S1P) receptors and levels, small GTPases, kynurenine pathway products, tyrosine receptor kinase B (TrkB), purinergic signalling, exosomes, shifting between glycolysis and oxidative phosphorylation (OXPHOS), circadian gene dysregulation, and alterations in the melatonergic pathway regulation^[3,4]^. All of these changes have been linked to variations in mitochondrial function and the interactions of GBM/GSC with other cells in the tumour microenvironment^[4]^.

The current article highlights the role of the mitochondrial melatonergic pathway in GBM/GSC pathophysiology and tumour microenvironment interactions, including via the regulation of the circadian genes *CLOCK* and *Bmal1*, which modulate temozolomide efficacy^[5]^. First, the key factors and processes associated with GBM/GSC pathophysiology are reviewed and integrated into a model of GBM/GSC pathophysiology, into which chemoresistance can then be placed.

## GBM/GSC pathophysiology

### General

GBM/GSC show considerable pathophysiological heterogeneity^[6]^, driven by the dynamic intercellular interactions in the GBM/GSC microenvironment, indicating a complexity considerably greater than classical “go or grow” phenotypes. The involvement of the circadian rhythm and gut microbiome/permeability-driven changes in mitochondrial function^[7,8]^ indicate a role for systemic processes and provide a more holistic perspective on GBM/GSC pathophysiology, and thereby more diverse treatment targets.

### Mitochondrial melatonergic pathway

**Figure 1:**
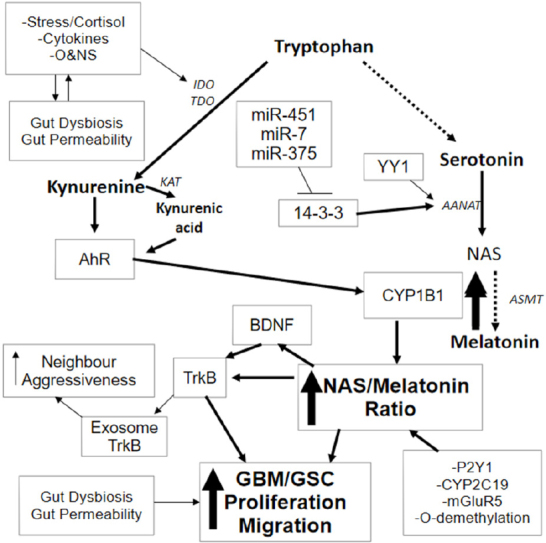
Stress, pro-inflammatory cytokines and O&NS can all increase IDO and/or TDO, leading to an increase in kynurenine and kynurenic acid, which are AhR ligands. AhR activation can drive significant changes in GBM/GSC, including an increase in CYP1B1 within mitochondria. Mitochondria CYP1B1 “backward” convert melatonin to NAS. NAS activates TrkB directly as well as via BDNF induction, leading to GBM/GSC proliferation and survival. A number of other factors and processes can increase the NAS/melatonin ratio, including CYP2C19, P2Y1 receptor, mGluR5 receptor and *O*-demethylation. These also provide trophic support to GSC and contribute to chemoresistance. The mitochondrial melatonergic pathway can also be regulated by miR-7, miR-375 and miR-451, which inhibit 14-3-3, thereby decreasing the 14-3-3 stabilization of AANAT, and inhibit both NAS and melatonin production. TrkB can also be released in the GBM/GSC exosomes, leading to an increase in aggressiveness of neighbouring cells. The upregulation of YY1 in GBM/GSC can increase melatonergic pathway activation. Stress, inflammation and O&NS can also induce gut dysbiosis and increase gut permeability, with the resultant decrease in butyrate contributing to GBM/GSC pathoaetiology and pathophysiology. Evolution has provided a number of ways to regulate the initiation and products of the melatonergic pathway. AANAT: aralkylamine *N*-acetyltransferase; AhR: aryl hydrocarbon receptor; ASMT: acetylserotonin methyltransferase; BDNF: brain derived neurotrophic factor; CYP: cytochrome P450; GBM: glioblastoma multiforme; GSC: glioblastoma stem-like cells; IDO: indoleamine 2,3-dioxygenase; KAT: kynurenine aminotransferase; mGluR: metabotropic glutamate receptor; NAS: *N*-acetylserotonin; O&NS: oxidative and nitrosative stress; P2Y1: purinergic receptor; TDO: tryptophan 2,3-dioxygenase; TrkB: tyrosine receptor kinase B

miRNAs are an important aspect of epigenetic regulation, given the ability of individulual miRNAs to coordinate up to 100 genes. Consequently, changes in miRNAs are important for coordinating plasticity responses in cells, including GBM/GSC. Numerous miRNAs show changes in GBM/GSC, including miR-7, miR-375 and miR-451^[9-11]^. These three miRNAs suppress 14-3-3ζ protein levels, thereby preventing 14-3-3ζ from stabilizing aralkylamine *N*-acetyltransferase (AANAT). AANAT is the initial enzyme of the melatonergic pathway^[12]^. Heightened 14-3-3 protein levels are evident in GSC^[13]^, which correlates with poor survival rates and chemoresistance^[14]^. The 14-3-3ζ stabilization of AANAT drives the conversion of serotonin to *N*-acetylserotonin (NAS). NAS can then be converted to melatonin by acetylserotonin methyltransferase (ASMT). However, a number of cellular processes can ‘backward’ convert melatonin to NAS, including *O*-demethylation; ATP activation of the purinergic receptor, P2Y1; glutamate activation of the metabotropic glutamate receptor (mGluR) 5; cytochrome P450 (CYP) 2C19; and the induction of CYP1B1, reviewed in^[4]^. This allows such cellular processes to drive significant variations in the NAS/melatonin ratio. This is of some significance as the melatonergic pathway is evident in every body cell, including within mitochondria. This may be of importance to GBM/GSC pathophysiology as melatonin induces GBM/GSC apoptosis, whilst NAS, via activation of the brain-derived neurotrophic factor (BDNF) receptor, TrkB, increases GSC survival and proliferation^[15]^. Consequently, the roles of miR-7, miR-375, miR-451 and 14-3-3ζ in GBM/GSC pathophysiology and treatment resistance will include modulation of the mitochondrial melatonergic pathway. This also suggests that factors that act to suppress the AhR, including resveratrol, may be mediating efficacy, at least partly, by decreasing the NAS/melatonin ratio, as reviewed in^[4]^. Variations in the NAS/melatonin ratio will also be relevant to data showing TrkB within GBM/GSC exosomes for transferring aggressiveness to neighbouring GBM^[16]^ [Figure 1].

The AhR is classically known as the dioxin receptor, although accumulating evidence shows the AhR to have a number of exogenous, induced and endogenous ligands, with some differential effects that are specific to different ligands. As such, the AhR is an important aspect of cellular plasticity. AhR-induced CYP1B1 may be of particular relevance to the backward conversion of melatonin to NAS. The AhR is also a known modulator of GBM/GSC physiology^[17]^. As noted, the AhR has many endogenous and exogenous ligands, including kynurenine and kynurenic acid, both of which are induced downstream of pro-inflammatory cytokine- and stress-induced indoleamine 2,3-dioxygenase (IDO) and tryptophan 2,3-dioxygenase (TDO). The AhR may therefore allow stress and systemic processes to regulate GBM/GSC pathophysiology via an increase in the NAS/melatonin ratio [Figure 1]. The relevance of AhR activation by kynurenine and kynurenic acid requires investigation^[18]^, given the trophic support to GSC provided by the CYP1B1 driven increase in the NAS/melatonin ratio. Temozolomide increases IDO1 in GBM cultures, thereby potentially increasing GSC trophic support via kynurenine activated AhR and increased NAS activation of TrkB^[19]^. This could be one mechanism whereby temozolomide contributes to its own chemoresistance.

### Circadian regulation of mitochondrial metabolism

Alterations in mitochondrial function, both in GBM/GSC and other tumour microenvironment cells, are an important aspect of GBM/GSC pathophysiology. Interactions between cells may be seen as a form of mitochondria-to-mitochondria communication given the powerful influence that these organelles have in co-ordinating cellular and intercellular processes. The circadian rhythm is a powerful regulator of mitochondrial function, as highlighted in immune cells, where night-time, pineal gland-derived melatonin acts to dampen and reset mitochondrial metabolism in immune cells to OXPHOS, thereby dampening any immune cell activation. Immune cell activation occurs in association with glycolytic metabolism. The immune-dampening effects of melatonin are mediated via its activation of the circadian gene, *Bmal1*. *Bmal1*, likely via the inhibition of pyruvate dehydrogenase kinase (PDK), disinhibits the pyruvate dehydrogenase complex (PDC), allowing pyruvate to be converted to acetyl-CoA. Acetyl-CoA upregulates ATP production by the tricarboxylic acid (TCA) cycle and OXPHOS, and is a necessary co-substrate for AANAT and the mitochondrial melatonergic pathway^[8,20]^. Such circadian switching of mitochondrial metabolism in immune cells parallels the dynamic shifts in GBM/GSC metabolism, with OXPHOS associating with proliferation, whilst cytosolic glycolysis is predominant during migration^[9]^. Alterations in the circadian-driven switching between OXPHOS and glycolysis are proposed to contribute to cancer pathoaetiology^[8,20]^.

Many factors can act to modulate the circadian influence on mitochondrial function. Clearly, dampening the immune response at night is not optimal when an immune response to a challenge is required. The increase in pro-inflammatory cytokines that are evident during an immune challenge act to switch off pineal melatonin production, thereby preventing the melatonin-Bmal1-PDC path from inducing OXPHOS and immune cell quiescence^[21]^. Notably, circadian factors have been relatively little investigated in GBM/GSC and tumour microenvironment cells, contributing to the heterogeneity in GBM/GSC pathophysiology in published studies, including in regard to the AhR^[18]^. All cells are subject to powerful circadian regulation.

### Gut microbiome, gut permeability and GBM/GSC

The dysregulation of the gut microbiome, gut dysbiosis, and increased gut permeability all play important roles in the biological underpinnings of a wide array of diverse medical conditions including cancer^[22,23]^. A number of factors, including stress, diet, lifestyle, pro-inflammatory cytokines and oxidative stress can increase gut permeability, whilst concurrently contributing to gut dysbiosis. Gut dysbiosis is often associated with a decrease in the gut microbiome-derived short-chain fatty acid, butyrate. Butyrate not only helps maintain the integrity of the gut barrier, but also enters the general circulation where it acts to dampen immune and glial cell inflammatory activity. This is achieved primarily via butyrate’s capacity as a HDAC inhibitor and its ability to optimize mitochondrial function via PDC disinhibition and the upregulation of acetyl-CoA, the TCA cycle, OXPHOS and the associated induction of the melatonergic pathway^[7]^. It is by such mechanisms that gut-derived butyrate modulates immune cell function, thereby allowing the gut to modulate a host of diverse medical conditions.

Butyrate, including via its nutriceutical salt form, sodium butyrate, induces apoptosis in GSC^[24]^ as well as increasing the senescence and apoptotic susceptibility of GBM^[25]^, indicating a role for decreased gut microbiome-derived butyrate in the pathoaetiology and pathophysiology of GBM/GSC. The induction of gut dysbiosis by stress, obesity and dietary factors allow these factors to have a role in GBM/GSC pathoaetiology and pathophysiology. Alcohol intake correlates positively with GBM/GSC risk^[26]^, with high alcohol intake/binge drinking strongly associated with gut dysbiosis/permeability and decreased butyrate^[27]^. Whether the association of alcohol intake with GBM/GSC risk is mediated via gut dysbiosis/permeability will be important to determine. Butyrate not only directly modulates GBM/GSC, but also does so for other cells in the GBM microenvironment, including microglia and macrophages^[28]^, which comprise up to 40% of the GBM/GSC microenvironment^[29]^. Consequently, variations in butyrate may not only change interactions of the cells in the GBM/GSC microenvironment, but also provide a possible treatment option (see Treatment Implications section below).

Gut microbiome-derived butyrate may also be relevant to levels of mutant *p53*. Mutant *p53* leads to amyloid-like oligomers that readily shift to being oncogenic, in association with an increase in chemoresistance^[30]^, whilst also inhibiting the apoptotic effects of wild-type *p53*. Interestingly, sodium butyrate and other HDAC inhibitors can decrease the transcription of mutant *p53*, as shown in breast cancer cells, thereby making these cells more susceptible to chemotherapy^[31]^. This requires investigation in GBM/GSC as it may suggest a role for sodium butyrate in suppressing chemoresistance.

Mast cells line most epithelial surfaces and are classically associated with histamine production and allergic responses. Mast cells are also significant regulators of gut barrier homeostasis^[32]^. Stress-induced hypothalamic and amygdala corticotropin releasing hormone induces tumour necrosis factor alpha (TNF-α) by mucosal mast cells, thereby increasing gut permeability/dysbiosis^[33]^. Gut microbiome-derived butyrate, via HDAC inhibition, dampens mast cell activation^[34]^, allowing gut-derived butyrate to modulate the effects of stress. Mast cells may also be significant treatment targets in GBM/GSC as their chemoattraction to the tumour microenvironment is associated with improved patient survival (see Treatment Implications section below).

Heightened levels of gut permeability also have significant systemic consequences via elevations in circulating lipopolysaccharide (LPS) levels and the release of exosomal high mobility group box (HMGB)1 from intestinal epithelial cells^[7,35]^. Exosomes are released by most cells and contain variable content that are then released when the exosome “bubble” merges with the plasma membrane of another cell. Exosomes are increasingly being recognized as an important aspect of intercellular communication, including via their miRNA content. Both LPS and exosome HMGB1 can activate the toll-like receptor-4 (TLR-4), with both HMGB1^[36]^ and LPS^[37]^ modulating GBM/GSC and the GBM/GSC microenvironment. This may be relevant to a role of central inflammatory processes in GBM/GSC aetiology, as gut-derived LPS and HMGB1, via microglia TLR activation, raise levels of superoxide and inducible nitric oxide synthase, which readily form peroxynitrite and thereby, acidic sphingomyelinase and ceramide. Although ceramide is able to induce GBM/GSC apoptosis, its role in the inflammatory aetiology of GBM/GSC requires investigation as it readily suppresses mitochondrial function, including by decreasing 14-3-3 and therefore the mitochondrial melatonergic pathway^[38]^. This is another route whereby alterations in the gut may have a role in GBM/GSC pathoaetiology.

It is by such processes that gut dysbiosis/permeability can impact upon GBM/GSC and tumour microenvironment cells, with relevance both to the pathoaetiology and pathophysiology of GBM/GSC, as well as to chemoresistance that is commonly evident. As butyrate induces the melatonergic pathway^[39]^, the impact of such gut processes may be mediated via the regulation of the mitochondrial melatonergic pathway.

### Wider GBM/GSC pathophysiology

Other common GBM/GSC pathophysiological factors may also be linked to variations in the regulation of the mitochondrial melatonergic pathway, including an array of miRNAs.

### microRNAs

Epigenetic regulation of the genome by microRNAs is a significant determinant of patterned gene expression, with each miRNA able to regulate up to 100 genes. Many miRNAs are altered in GBM, with over 250 being upregulated and around 100 downregulated^[11,40]^. A number of miRNAs are correlated with GBM/GSC chemoresistance and radio-resistance^[40]^. However, it should be noted that most miRNA investigations have failed to look at their relevance to the heterogeneity in GBM/GSC, including the differences between GBM and GSC. As many miRNAs are differentially regulated over the circadian rhythm^[41]^, as well as being significant modulators of the circadian rhythm and circadian genes^[42]^, variations in miRNAs are also likely to be evident over the circadian rhythm and during the dynamic interactions of cells in the tumour microenvironment. Snapshots of miRNA changes in GBM/GSC will not reflect these dynamic shifts, including in mitochondrial function.

### miR-155/GRP78/CD44

Hypoxia-inducible factor (HIF) can increase stem cell-like features, at least partly driven by an increase in glucose-regulated protein (GRP) 78 and GRP78-induced CD44, with effects linked to an increase in the PI3K-Akt-mTOR pathway^[43,44]^. Although GRP78 normally acts as an endoplasmic reticulum chaperone, its expression on the plasma membrane mediates its pro-stem cell effects. Recent data shows CD44 to be increased in GBM and contribute to a GSC-like phenotype, in association with poor patient outcomes^[45]^. An upstream increase in miR-155 can induce the GRP78-CD44 path and stemness, as shown in breast cancer stem-like cells^[46]^. Interestingly, the miR155 host gene (*miR155HG*) is a lncRNA that acts to sponge miR-185^[47]^ and thereby, enhance annexin (ANX) A2 levels. ANXA2 can then provide feedback to elevate miR155HG levels via the phosphorylation of signal transducer and activator of transcription (STAT) 3, which binds to the miR155HG promoter^[47]^. This would suggest a positive feedback loop on miR155HG levels, with concurrent increases in miR-155 contributing to the induction of stemness via GRP78-CD44. A role for decreased miR-185 in GBM/GSC pathophysiology is supported by data showing poorer outcomes in GBM patients with lower levels of circulating miR-185^[48]^. Elevations in ANXA2 are associated with an increase in angiogenesis and proliferation in GBM patients^[49]^, and thus, with an increase in chemoresistance^[50]^.

In non-neoplastic cells, melatonin decreases GRP78 levels in stressed cells via impacts on mitochondrial function^[51]^, with melatonin inhibiting an array of different cancer stem cells typified by high GRP78-CD44 expression^[52,53]^. In colorectal cancer stem cells, there is a negative correlation between CD44 and the melatonergic pathway^[54]^. Such data clearly highlight the need for future research to clarify the nature and relevance of the melatonergic pathway in GBM/GSC, especially as previous data shows melatonin’s utility in the induction of GSC apoptosis^[55]^. It should be noted that a number of studies show melatonin inhibits GSC survival and proliferation, with effects that are mediated by a number of different signalling pathways^[55-58]^. It is also of note that a number of the miRNAs showing alterations in GBM/GSC can act to regulate the melatonergic pathway, including miR-7, miR-375 and miR-451, suggesting that the mitochondrial melatonergic pathway may be a relevant aspect of the dynamic interactions within the GBM/GSC microenvironment.

### miR-451

A number of studies show miR-451 alterations in GBM/GSC^[59,60]^. miR-451 seems important to the shifting between proliferation and migration, which the authors propose to be driven by microenvironment cell modulation of GBM/GSC^[9]^. Such work highlight how the cells of the microenvironment may contribute to the heterogeneity and plasticity of GBM/GSC. Other factors associated with GBM/GSC pathophysiology and heterogeneity may also be regulated by variations in miR-451 levels, including the AMPK-mTOR pathway, 14-3-3 proteins, and the small GTPase, Rac1^[4]^. Importantly, miR-451 represses 14-3-3ζ protein levels, indicating a role in the inhibition of the melatonergic pathway, and suggesting that its variable expression under proliferation, versus metastasis, may be reflective of changes in the regulation of mitochondrial function by the melatonergic pathway^[9]^.

### miR-7

GBM/GSC migration and proliferation can be inhibited by miR-7-5p, which also decreases chemoresistance. These effects of miR-7-5p are via its down-regulation of the transcription factor, yin yang (YY) 1^[10]^. YY1 is a significant positive regulator of the melatonergic pathway, as shown in the retina^[61]^. miR-7 inhibits NAS and melatonin synthesis, as well as downregulating 14-3-3ζ in GBM cell lines^[62]^. miR-7 also modulates mitochondrial metabolism in other cell types^[63]^. As such, the roles of miR-7-5p in GBM/GSC include regulation of the melatonergic pathway.

### miR-375

Decreased miR-375 levels are evident in GBM/GSC tissue, as well as in GBM cell lines, with the over-expression of miR-375 inhibiting both the proliferation and migration of GBM^[11]^. Like miR-7, miR-375 also downregulates 14-3-3ζ, indicating that it will act to suppress the stabilization of AANAT and thereby inhibit initiation of the melatonergic pathway. It is unknown whether miR-375 modulates YY1 expression.

### 14-3-3 proteins

14-3-3 proteins are often in complex with many other cellular factors, including in their role as chaperones. Alterations in 14-3-3 proteins can therefore have significant impact on cellular function. Elevations in a number of different 14-3-3 protein isoforms are associated with resistance to apoptosis in GBM/GSC and poorer patient survival rates. 14-3-3ζ downregulation sensitizes GBM cells to apoptosis induction^[64]^, with elevations in 14-3-3ζ levels correlating with glioma grade, being very high in GSC^[65]^, as reviewed in^[4]^. Such data highlights the significance of 14-3-3 protein regulation by miRNAs. The downregulation of miR-7, miR-375 and miR-451 in GBM/GSC would have many consequences, including the upregulation of 14-3-3, and thereby AANAT stabilization. This may be most parsimoniously associated with an increase in the NAS/melatonin ratio, suggesting that the increase in 14-3-3 and alterations in miR-7, miR-375 and miR-451 will be co-ordinated with an increase in the factors and processes that ‘backward’ convert melatonin to NAS. Increased NAS, via TrkB activation, will contribute to GBM/GSC survival and proliferation, as well as transferring aggressiveness to neighbouring cells when carried within exosomes [Figure 1].

### YY1

The transcription factor, YY1, is a significant promoter of cancer and chemoresistance, including in GBM/GSC, although it can have some antitumour effects^[66]^. YY1 inhibition in tumours has a number of consequences, including decreasing mutant p53 levels^[31]^ and thereby suppressing mutant p53-induced chemoresistance^[30]^. Whether elevated YY1 levels are co-ordinated with NAS induction and TrkB activation in GSC requires investigation. HIF-1 directly regulates TrkB in tumour cells^[67]^. This requires investigation in GBM/GSC, including whether TrkB induction is co-ordinated with an increase in YY1, and the “backward” conversion of melatonin to NAS by CYP1B1, mGluR5, P2Y1 receptor, CYP2C19 and/or *O*-demethylation. Variations in the regulation of the NAS/melatonin ratio may be a significant determinant of the sometimes-contrasting effects of YY1 on tumourigenesis^[66]^.

Importantly, YY1 regulates circadian genes and the circadian rhythm. YY1 induces miR-135b, thereby suppressing Bmal1 and increasing tumourigenicity^[68]^. This is proposed to impair the local circadian gating control of tumour suppression, thereby increasing tumourigenesis and chemoresistance. YY1 may therefore co-ordinate circadian dysregulation, elevate mutant p53 and NAS activation of TrkB in GSC. Bmal1 is the major mediator of the circadian “resetting” of mitochondrial metabolism via PDK inhibition and associated PDC disinhibition. Consequently, the YY1-miR-135b suppression of Bmal1 is likely to be an important aspect of how alterations in mitochondrial metabolism occur in the tumour microenvironment. A number of miRNAs suppress YY1 and thereby, prevent stemness and temozolomide resistance in GBM/GSC, including miR-7-5p^[10]^ and miRNA-186^[69]^.

Interestingly, YY1 is essential for normal stem cell renewal via the regulation of mitochondrial metabolism^[70]^. The increase in mTOR that is evident in aggressive tumours, including GBM/GSC, regulates mitochondrial metabolism and cell proliferation via YY1 and YY1-induced peroxisome proliferator-activated receptor γ, coactivator 1α (PGC-1α)^[71]^, thereby contributing to heightened levels of glucose uptake and utilization^[72]^. In non-neoplastic B-cells, YY1 directly binds to over 20 mitochondrial function-related genes^[73]^. YY1 is also an important regulator of mitochondrial metabolism across an array of different cancers^[74]^, highlighting the importance of tumour YY1 in driving the alterations in mitochondrial communication across the different cells of the tumour microenvironment. As sodium butyrate suppresses the transcriptional activity of YY1^[31]^, the adjunctive use of sodium butyrate may have utility in GBM/GSC management. This may also indicate a role for factors inducing gut dysbiosis in the aetiology of GBM/GSC, including from prolonged alcohol abuse^[26]^.

Overall, YY1 seems an important aspect of GBM/GSC pathophysiology and chemoresistance, with effects via the regulation of mitochondrial metabolism and the melatonergic pathway. Given that the YY1 induction of miR-135b suppresses Bmal1, as shown in pancreatic cancers^[68]^, YY1 may be important to the loss of circadian regulation of metabolism that is proposed to underpin tumour aetiology^[8,20]^ as well as ongoing tumour pathophysiology. Higher levels of Bmal1 correlate with improved patient survival across different tumours^[75]^. As such, YY1, like the miRNAs reviewed above, may mediate its effects in GBM/GSC via processes that include alterations in the circadian regulation of GBM/GSC, in part via changes in the mitochondrial melatonergic pathway.

## Tumour microenviroment

Clearly, the interactions of GBM/GSC with cells in the tumour microenvironment are crucial for understanding GBM/GSC pathophysiology and treatment resistance. The melatonergic pathway would seem an intimate aspect of the intercellular interactions in the tumour microenvironment.

### Kynurenine and AhR

As noted, AhR activation is one of the factors that may contribute to increasing the NAS/melatonin ratio. GBM can release kynurenine, thereby inducing autocrine and paracrine activation of the AhR. AhR activation in macrophages suppresses NF-κB^[76]^. Interestingly, NF-κB not only transiently induces macrophage activation, but also induces processes that then shift macrophages to an M2-like, pro-phagocytic phenotype, which is mediated by the autocrine/paracrine effects of released melatonin^[77]^. As such, the kynurenine pathway and AhR activation may be another aspect of GBM/GSC modulation of the tumour microenvironment, with effects that include not only alterations in macrophage phenotype and metabolic state, but also a decrease in the production of melatonin in the tumour microenvironment. The GBM release of kynurenine may be another mechanism whereby GBM/GSC influence cells in the tumour microenvironment via alterations in the mitochondrial melatonergic pathway.

### Microglia and macrophages

Microglia and macrophages can form up to 40% of the cells within the tumour microenvironment and have bidirectional interactions with GBM/GSC^[29]^. Microglia and macrophages can release growth factors and cytokines that favour GBM/GSC survival and proliferation^[78]^. Incorporating microglia into GBM cultures increases GBM survival and confers chemoresistance^[79]^. However, this is dependent on the “phenotypic state” of microglia, which can vary according to location in the tumour microenvironment. Elevation in, and release of, a subset of GBM let7 miRNAs activates microglia TLR7, leading to an increase in pro-inflammatory cytokines and the suppression of GBM growth^[80]^. This requires further investigation, including what regulates specific let7 miRNA subtypes, and whether their release is via exosomes. This highlights another aspect of miRNA function *viz*-*a-viz* ligands to TLRs. Overall, microglia and macrophages in the tumour microenvironment are in intimate two-way interaction with GBM/GSC, with consequences for GBM/GSC survival, migration and proliferation.

Clearly, such interactions of GBM/GSC with microglia and macrophages will involve alterations in mitochondrial function across cell types. Given that immune cells have their mitochondrial metabolism regulated by the circadian rhythm, primarily melatonin-Bmal1 induced OXPHOS, it requires investigation as to how relevant such circadian processes are to the interactions of these cells with GBM/GSC, including how circadian rhythms and circadian gene regulation are co-ordinated between GBM/GSC and microglia/macrophages. As indicated above, the induction of Bmal1 by melatonin disinhibits PDC, leading to an increased conversion of pyruvate to acetyl-CoA, thereby increasing OXPHOS, the TCA cycle and the mitochondrial melatonergic pathway. In macrophages/microglia this would induce a quiescent state. As noted, GBM can activate the AhR on neighbouring cells via the release of kynurenine, thereby increasing AhR-induced CYP1B1 and the “backward” conversion of melatonin to NAS. This is one mechanism whereby GBM/GSC can act to regulate microglia and macrophage metabolism, concurrent to increasing NAS production for the activation and proliferation of GSC. The relevance of the circadian rhythm and alterations in circadian genes on the interactions of GBM/GSC with microglia/macrophages and other cells in the microenvironment such as astrocytes clearly requires further investigation, given the impact that this can have on GBM/GSC pathophysiology and therefore, chemoresistance.

### Endothelial cells

Other cells in the tumour microenvironment that are important to GBM/GSC survival and chemoresistance include endothelial cells. β-catenin/Wnt-mediated transformation of endothelial cells into mesenchymal stem cell-like cells increases chemoresistance to temozolomide in a GBM preclinical model^[81]^, highlighting the role of non-immune cells in the tumour microenvironment in GBM/GSC chemoresistance. Controlling the melatonergic pathway may be of some importance to this as melatonin induces the differentiation of mesenchymal stem cells^[82]^ whilst TrkB activation is a major driver of the epithelial-mesenchymal transition across a range of different cancers^[83]^. TrkB activation can also activate the β-catenin/Wnt pathway^[84]^, suggesting a role for NAS activation of TrkB in the transition of endothelial cells to mesenchymal stem cell-like cells^[81]^. This is another mechanism whereby the local NAS/melatonin ratio will have contrasting effects on GBM/GSC, including on chemoresistance.

### Astrocytes

Astrocyte-like neuronal stem cells carrying driver mutations are proposed to be the GBM/GSC cells of origin, following their migration from the subventricular zone into other brain regions^[85]^. There is a growing interest in the role of GBM/GSC-associated astrocytes in the tumour microenvironment, with tumour-activated astrocytes modulating GBM/GSC proliferation as well as radio- and chemo-resistance^[86]^. In a human organotypic slice model incorporating astrocytes, microglia and GBM, the activation of the Janus Kinase (JAK)/STAT pathway in astrocytes leads to the release of a number of anti-inflammatory factors, including IL-10, transforming growth factor β and granulocyte-colony stimulating factor^[87]^. This parallels the release of these factors in GBM/GSC, which are proposed to induce an immunosuppressive tumour microenvironment^[88]^. The inhibition of the astrocyte JAK/STAT pathway shifts astrocytes to the production of pro-inflammatory cytokines in the GBM microenvironment^[87]^, suggesting that the modulation of astrocyte function will contribute to the plasticity of responses in the GBM/GSC microenvironment.

A wide array of astrocytic factors are associated with GBM/GSC progression, including the release of glutamate via the cystine/glutamate antiporter system, Xc-^[89]^, as well as by processes that involve cell-to-cell contact^[90]^. Glutamate activation of mGluR5 can increase CYP1B1 and thereby the NAS/melatonin ratio. Astrocytes may be a significant source of NAS and melatonin, with the YY1 that is increased in reactive astrocytes being a potential driver of the astrocyte melatonergic pathway^[91]^. Factors that act to regulate the NAS/melatonin ratio, including P2Y1 receptor, Ahr/CYP1B1, CYP2C19, mGluR5 and *O*-demethylation processes are all evident in astrocytes, suggesting that astrocytes will be a potential source of NAS for TrkB activation in GSC. Overall, the role of astrocytes, like macrophages and microglia, in the GBM/GSC microenvironment can include variations in the NAS/melatonin ratio, with differential consequences for GBM/GSC survival and proliferation [Figure 1].

## Ceramide, S1P and estrogen

**Figure 2:**
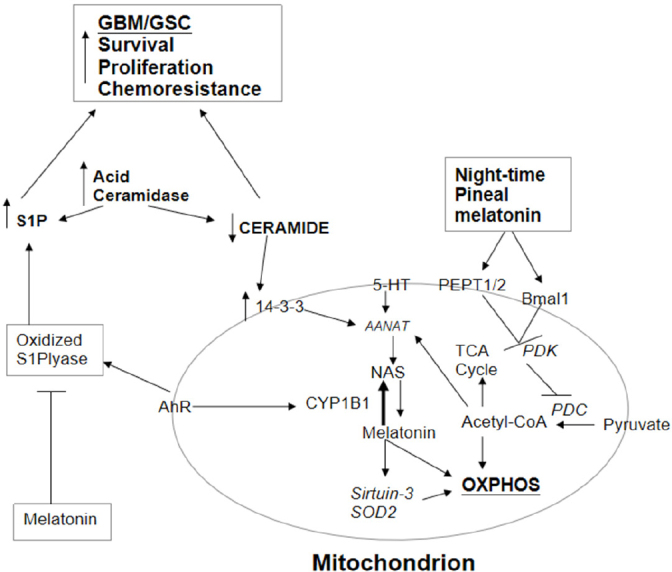
AhR activation not only increases CYP1B1 and the NAS/melatonin ratio but also oxidizes S1P lyase, leading to an increase in S1P. S1P is increased and ceramide decreased by the elevation in acid ceramidase in GBM/GSC. The increase in the S1P/ceramide ratio contributes to GBM/GSC survival, proliferation, migration and chemoresistance. The decrease in ceramide attenuates its suppression of 14-3-3, thereby potentiating the mitochondrial melatonergic pathway. Pineal melatonin is taken up by PEPT1/2 into mitochondria and induces Bmal1, which inhibits PDK, leading to disinhibition of the PDC, resulting in increased conversion of pyruvate to acetyl-CoA. Acetyl-CoA increases ATP from the TCA cycle and OXPHOS, as well as being a necessary co-substrate for AANAT and melatonergic pathway activation. Melatonin is intimately linked with the regulation of sirtuins and with antioxidant enzymes, including SOD2. Melatonin also inhibits the oxidized S1P lyase, leading to a decrease in S1P oncogenic effects. It is by such processes that variations in the mitochondrial melatonergic pathway are intimately linked to the intra- and extracellular processes in GBM/GSC and the cells of the tumour microenvironment. AANAT: aralkylamine *N*-acetyltransferase; AhR: aryl hydrocarbon receptor; ASMT: acetylserotonin methyltransferase; CYP: cytochrome P450; GBM: glioblastoma multiforme; GSC: glioblastoma stem-like cells; NAS: *N*-acetylserotonin; OXPHOS: oxidative phosphorylation; PDC: pyruvate dehydrogenase complex; PDK: pyruvate dehydrogenase kinase; PEPT: peptide transporter; S1P: sphingosine-1-phosphate; SOD: superoxide dismutase; TCA: tricarboxylic acid

The last couple of years have shown a resurgence in interest in acid ceramidase inhibitors in the management of GBM/GSC. This was first indicated in a study by Hara and colleagues in 2004, where they showed the effects of radiotherapy to significantly interact with the expression of functional p53 in the regulation of acid ceramidase and aSMase^[92]^. Recent work has supported the role of elevations in acid ceramidase in contributing to radio-and chemo-resistance in GBM/GSC^[93]^, and perhaps, especially in GSC^[94]^. These authors show that the inhibition of acid ceramidase leads to an increase in ceramide that drives apoptosis in patient-derived GSC^[94]^. As temozolomide has no effect on acid ceramidase, this is a potentially novel treatment target. The pro-apoptotic effects of ceramide include the suppression of 14-3-3 levels^[38]^, indicating that ceramide will suppress the stabilization of AANAT, and therefore suppress the melatonergic pathway. As such, the efficacy of acid ceramidase inhibitors will include the suppression of the mitochondrial melatonergic pathway, thereby inhibiting mitochondrial function and GBM/GSC plasticity. See Figure 2.

The efficacy of tamoxifen in a range of cancers, including to some degree in GBM/GSC, is proposed to be mediated by acid ceramidase inhibition^[95]^, indicating a novel pathway for tamoxifen in the management of GBM/GSC. The estrogen receptor (ER) α36 variant is expressed in GBM and not only attenuates tamoxifen efficacy via heightened autophagy but is markedly increased in tamoxifen resistant GBM cells, suggesting a role for ERα36 in chemoresistance^[96]^. The high levels of the classical ERα and relatively low ERβ in GBM/GSC can increase proliferation, indicating some tamoxifen efficacy via its inhibition of ERα signalling^[97]^. Although a range of neurosteroids are produced in GBM/GSC, their relevance to GBM/GSC pathophysiology requires clarification^[98]^. Increased levels of local estrogen production can increase AhR-induced CYP1A/B1, thereby elevating the NAS/melatonin ratio. Melatonin is a significant inhibitor of ERα, and therefore of ERα activation of the AhR. This is another means by which melatonin may modulate treatment-resistance in GBM/GSC.

In ERα-positive breast cancer, tamoxifen resistance is associated with AhR activation^[99]^, whilst its efficacy is modulated by CYP2C19 alleles^[100]^. The relevance of the AhR and CYP2C19 alleles to tamoxifen effects in GBM/GSC requires investigation, including whether these factors are acting via the NAS/melatonin ratio. Tamoxifen inhibits mitochondrial Complex I activity in GBM/GSC, possibly indicative of more direct effects on mitochondrial function^[101]^. Whether melatonin’s inhibition of the AhR and ERα have significant impact via the NAS/melatonin ratio will be important to determine, as this could suggest that melatonin would have adjunctive utility with tamoxifen treatment^[102]^. Overall, the efficacy of tamoxifen in GBM/GSC may be associated not only with ERα, AhR and NAS/melatonin regulation, but also with its inhibition of acid ceramidase. An important consequence of acid ceramidase inhibition is downregulation of the S1P pathway.

A decrease in sphingosine and its conversion to S1P is a downstream consequence of acid ceramidase inhibition and elevation in ceramide. The attenuation in S1P production is relevant to the pro-apoptotic effects of acid ceramidase inhibitors, with S1P associated with GBM/GSC survival, migration, and proliferation as well as angiogenesis^[4]^ [Figure 2]. The S1P receptor subtypes, and their differential induction of small GTPases, primarily S1P1 receptor-induced Rac-1 and S1P2/3 receptor-induced RhoA, are integral to cellular plasticity, including migration and proliferation in GBM/GSC^[103,104]^. This has parallels to the plasticity associated with neurogenesis^[105]^, which seems the initial source of GSC^[85]^. The S1P receptor subtypes are important drivers of core features of the cellular phenotype, including mediating migratory processes. This is likely to be driven by plasma membrane changes in the organization of lipid rafts, with the inhibition of cholesterol synthesis and uptake that suppresses GBM/GSC survival, leading to alterations in S1P receptor subtypes in lipid rafts^[106]^. S1P is also a significant regulator of the migration and phenotype of immune and stem cells, and is therefore likely to be an important aspect in GSC formation as well as the intercellular interactions of the tumour microenvironment^[107]^.

S1P is also an important regulator of immune cell mitochondrial function and metabolism^[108]^. This is not surprising, given that variations in S1P receptor subtype signalling are important in driving migratory *vs.* proliferative phenotypes, which are also intimately regulated by variations in mitochondrial metabolism. Fluxes of S1P between astrocytes and neuronal stem cells seem crucial in neurogenesis^[105]^ and the role of S1P and its receptor subtypes in intercellular interactions within the GBM/GSC microenvironment require further investigation, including how the ceramide/S1P ratio interacts with the melatonergic pathway. As data in non-neoplastic cells indicates interactions of Rac1, RhoA/ROCK with PDK^[109]^, it is not unlikely that the S1P receptor subtypes and their downstream small GTPases are intimately linked to the regulation of PDC, and therefore, with acetyl-CoA for the Krebs cycle and mitochondrial melatonergic pathway. The S1P receptors induction of small GTPases are a significant treatment target in GBM/GSC^[110]^, and may be intimately linked to mitochondrial metabolism and melatonergic pathway regulation.

AhR activation in breast cancer stem cells increases proliferation via an elevation in sphingosine kinase 1 and S1P receptor activation^[111]^, driven by AhR-mediated oxidation of S1P lyase at residue 317^[112]^. As this would be co-ordinated with an increase in CYP1B1 and therefore, with an increase in the NAS/melatonin ratio, the oncogenic effects of AhR-induced S1P may be intimately linked to mitochondrial metabolism and the mitochondrial melatonergic pathway. Melatonin, via its antioxidant capacity, would prevent such AhR-driven suppression of S1P lyase and its associated increase in S1P. Clearly, this requires investigation in the GBM/GSC microenvironment.

Overall, there is increasing interest in the differential effects of ceramide and S1P in the regulation of mitochondrial function^[113]^, and this clearly requires investigation in GBM/GSC, including their contrasting impacts on PDC, OXPHOS and the mitochondrial melatonergic pathway.

## Other chemoresistance factors in GBM/GSC

### Aldehyde dehydrogenase

Relapse following radio/chemo-therapy is common in GBM and is proposed to be due to therapy resistance of GSC. Temozolomide mediates its limited efficacy, at least in part, via an increase in lipid peroxidation and oxidative stress, which increases aldehydes and aldehyde-induced cytotoxicity^[114]^. Such cytotoxicity is attenuated in GSC due to their high expression of the aldehyde metabolizing enzyme, aldehyde dehydrogenase (ALDH) 1A3^[114]^. ALDH1A isozymes are increased in many cancer stem cells, where they contribute to chemoresistance^[115]^, although it should be noted that there is significant heterogeneity in ALDH1A3 expression in GSC, including within individuals.

ALDH isoforms may all vary in cancer stem-like cells in association with the functional state of p53^[116]^, indicating a close association with apoptotic processes in these cells. ALDH1A3 can be epigenetically, transcriptionally and post-translationally regulated, including by deubiquitination^[117]^ and FOXD1^[118]^. Other data show ALDH1A3 to be negatively regulated by autophagy^[119]^ and positively regulated by Wnt/β-catenin signalling^[120]^. ALDH1A3 can also function as a retinaldehyde dehydrogenase, thereby inducing retinoic acid, which is necessary for the upregulation of tissue transglutaminase in chemo-resistant GSC^[121]^. ALDH1A3 is also important for the survival of other cancer stem-like cells^[122]^, with effects proposed to be mediated via positive regulation of mitochondrial metabolism^[123]^.

It is also of note that ALDH enzymes are regulated by the circadian rhythm, as shown in the murine brain and liver^[124]^, with Wnt10A positively regulating some ALDH isoforms in breast cancer stem cells^[125]^. There is a growing appreciation of the role of circadian genes in the regulation of GBM/GSC, including in the regulation of chemoresistance. The maximal toxicity of temozolomide is optimized by upregulation of the circadian gene, *Bmal1*^[5]^. Alterations in the circadian gene, *CLOCK*, are also evident in GBM/GSC in association with a decrease in miR-124 and heightened levels of NF-κB activity^[126]^. CLOCK is the heterodimeric partner of Bmal1. Downregulating *Bmal1* or *CLOCK* in GSCs leads to cell-cycle arrest and apoptosis^[127]^. It requires investigation as to whether the contrasting effects of Bmal1 in potentiating temozolomide efficacy whilst increasing apoptosis when knocked-out, are driven by differential mitochondrial melatonergic pathway activation.

Overall, the role of ALDH1 in GBM/GSC chemo-resistance may be intimately linked to alterations in mitochondrial metabolism and regulation of the circadian rhythm, with the melatonergic pathway intimately associated with both processes.

## Integrative model

Circadian regulation is a core aspect of mitochondrial function, which is particularly evident in immune cells where they can be daily switched from a reactive state associated with glycolysis to a quiescent state driven by OXPHOS^[128]^ and sirtuin (SIRT) 1, with Bmal1 and SIRT1 mutually inducing each other in non-neoplastic cells^[129]^. Bmal1 shapes mitochondrial metabolism via PDK inhibition and consequent PDC disinhibition, thereby contributing to an increase in the conversion of pyruvate to acetyl-CoA. Acetyl-CoA increases the activation of the TCA cycle and OXPHOS, as well as acting as a necessary co-substrate for AANAT and thereby, mitochondrial melatonergic pathway activation. 14-3-3, especially the 14-3-3ζ isoform, is also important given its stabilization of AANAT ^[8]^. Such shifts in mitochondrial metabolism have been investigated primarily in immune cells, and form the basis of the immune-pineal axis^[21]^, whereby pineal melatonin induces the mitochondrial melatonergic pathway and OXPHOS in immune cells, leading to their night-time quiescence.

There is a growing realization that such shifts in mitochondrial metabolism are an inherent aspect of GBM/GSC plasticity, with glycolysis forming the metabolic underpinnings of a migratory phenotype, whilst OXPHOS may be associated with GSC survival and proliferation^[130]^. This is of some importance, given that OXPHOS inhibition leads to GSC apoptosis^[131]^. Such data would indicate that circadian genes, pineal melatonin, PDC and the mitochondrial melatonergic pathway might all be intimately linked to the regulation of GBM/GSC survival and phenotype. Importantly, such processes are also occurring in the other cells of the tumour microenvironment, including astrocytes, macrophages, endothelial cells and microglia. In this context, mitochondria may be seen as crucial nodes within cells, with control over the plethora of intercellular processes occurring between cells of the tumour microenvironment. The regulation of mitochondrial function across cell types would seem fundamental to the understanding of the tumour microenvironment, and therefore to GBM/GSC treatment. Mitochondria are where the power lies in cells, with interventions aimed at mitochondrial function acting in concert with core aspects of evolved cellular and intercellular control and function.

The mitochondrial melatonergic pathway is emerging as a powerful regulator of mitochondrial function, being intimately linked via PDC disinhibition and acetyl-CoA production to crucial aspects of mitochondrial metabolism. Exogenous melatonin decreases the survival, proliferation and metastasis of a wide array of different tumours, including GBM/GSC^[4]^, highlighting the dangers that GBM/GSC face in forming a microenvironment in which all cells can release melatonin, with an increase in mitochondrial melatonin being an integral part of their circadian regulation. Melatonin in tumour cells clearly has distinct effects from its beneficial effects in non-neoplastic cells. It is proposed that GBM/GSC overcome this problem by using the array of factors, including AhR/CYP1B 1, mGluR5, CYP2C19, P2Y1 receptor and *O*-demethylation to drive the backward conversion of melatonin to NAS. As well as limiting melatonin production, this also increases NAS availability for activation of the TrkB receptor, which has trophic effects in GSC. NAS also increases BDNF production in brain cells, indicating wider trophic effects arising from NAS release^[132]^. Importantly, these are not processes that are restricted to GBM/GSC plasticity, but are also important in the regulation of other cells within the tumour microenvironment, all of which potentially can efflux melatonin and/or NAS. As indicated, an array of mechanism can underpin this regulation of the melatonergic pathway across cell types. For example, the increase in glutamate levels in the tumour microenvironment has classically been associated with “space clearance” for GBM via excitotoxic damage. However, an increase in glutamate activation of mGluR5 can also increase the NAS/melatonin ratio. As such, much of the plasticity within the GBM/GSC microenvironment may be driven by processes that control mitochondrial metabolism and the mitochondrial melatonergic pathway. Mutations, epigenetic alterations and changes in the diverse array of receptors and intracellular signalling pathways may mediate their effects via their interactions with higher-order processes acting to regulate the mitochondrial melatonergic pathway.

Chemo-resistance may be seen in this context, whereby resistance to temozolomide or tamoxifen that often emerge following surgery for GBM/GSC may be seen as a mechanism in which GBM/GSC manage to maintain this control over the mitochondrial melatonergic pathway across different cells of the tumour microenvironment. This does not necessarily suggest total inhibition of melatonin production in the cells of the GBM/GSC microenvironment, but it does indicate that regulation of this pathway is of crucial importance. Future research should clarify the regulation of NAS and melatonin in different cell compartments. For example, melatonin uptake into mitochondria in oocytes is mediated by the peptide transporter (PEPT) 1/2^[133]^, the regulation of which may be crucial to the compartmental regulation of melatonin under specific conditions. Other changes in the cell may arise as a consequence of this, and possibly correlate with GBM/GSC maintenance, but would be arising from the need to regulate the mitochondrial melatonergic pathway and not because these other changes are crucial per se. Changes in circadian genes and the impact of the circadian rhythm may also be seen in this context. Such a model highlights the hierarchical influence of patterned transcriptions driven by mitochondrial function, and the key role that alterations of the mitochondrial melatonergic pathways have in driving such patterned changes.

As 14-3-3 stabilizes AANAT, the mitochondrial melatonergic pathway can also be regulated by 14-3-3 protein levels. 14-3-3 is expressed in mitochondria, with down-regulation of 14-3-3ζ induced by ceramide, sensitizing GBM to apoptosis, indicating a role for 14-3-3ζ in GBM/GSC chemoresistance^[65]^. Heightened expression levels of 14-3-3ζ in GBM patients are associated with a poor prognosis^[64]^. As to whether this is mediated by 14-3-3ζ stabilization of AANAT, leading to enhanced activation of the mitochondrial melatonergic pathway that is biased towards an increase in NAS production and TrkB activation requires investigation. A decrease in 14-3-3γ inhibits the stem-like qualities of GSC^[13]^, whilst the downregulation of 14-3-3β induces senescence in GBM/GSC^[134]^. Clearly, different 14-3-3 isoforms are important to GBM/GSC survival, which may be closely linked to 14-3-3 regulation of the mitochondrial melatonergic pathway.

Overall, the mitochondrial melatonergic pathway may be intimately linked to the plethora of diverse data associated with GBM/GSC pathophysiology. The melatonergic pathway has been evident from the beginning of cell evolution, being present in the first bacteria to become integrated into an early cellular structure that ultimately evolved into mitochondria^[135]^. Consequently, the melatonergic pathway has been an integral aspect of mitochondrial and cellular evolution and therefore, with the various mitochondrial factors, such as sirtuins, and processes such as metabolism, that underpin the function and survival of GBM/GSC and the cells of the tumour microenvironment.

## Futurel research

Does the increased YY1 in tumours associate with heightened levels of CYP1B1, mGluR5, P2Y1, *O*-demethylation and CYP2C19, and thereby with the “backward” conversion of melatonin to NAS and TrkB-driven GSC, survival and proliferation?

Does YY1-miR-135b inhibition of Bmal1 modulate OXPHOS in GBM/GSC over the circadian rhythm?

What is the influence of the circadian rhythm on gene expression in GBM/GSC, and in the cells of the tumour microenvironment, including miRNAs?

Do GBM/GSC determine the nature of the tumour microenvironment via the realignment of immune cell circadian rhythms and associated regulation of mitochondrial metabolism and the mitochondrial melatonergic pathway?

Does YY1-induced miR-135b regulate TNF receptor associated factor (TRAF) 2, given both miR-135b^[136]^ and TRAF2^[137]^ attenuate the transcriptional activity of Bmal1?

The AhR is an important regulator of the circadian rhythm, including by forming a heteromer with Bmal1, thereby inhibiting Bmal1’s influence on the circadian amplitude and metabolism^[138]^. AhR expression levels and activation over the circadian rhythm in GBM and GSC will therefore be important to determine.

As mGluR5 activation on microglia/macrophages can increase TrkB activation and BDNF production^[139]^, is this mediated via increased NAS?

As butyrate, via HDAC inhibition, modulates not only mast cell activation^[34]^ but increases GBM and GSC apoptosis^[24,25]^, do inducers of gut dysbiosis/permeability, such as stress, alcohol and diet, then impact on GBM/GSC pathoaetiology and pathophysiology via gut dysbiosis/permeability?

In some cells, sodium butyrate can increase AhR-induced induced CYP1^[140]^. This will be important to determine in GBM/GSC, especially the impact, if any, of sodium butyrate on CYP1B1-driven ‘backward’ conversion of melatonin to NAS over the circadian rhythm.

Do activated mast cells suppress GSC pathoaetiology, as indicated by the negative correlation of allergies and GBM development^[141]^?

Is there a role for mast cells in GBM/GSC pathophysiology, given mast cell suppression of GBM proliferation and migration, as well as their suppression of GSC self-renewal capacity and stemness markers^[142]^? What is the role of mast cell melatonin production^[143]^?

As wild-type p53 induces AhR-driven CYP1B1, does the resulting NAS suppress the apoptotic effects of wild-type p53 in GBM/GSC?

The roles of the ceramide/S1P ratio in the regulation of mitochondrial function, metabolism and melatonergic pathway in GBM/GSC and the cells of the tumour microenvironment will be important to determine.

Leucine zipper-EF-hand containing transmembrane protein (LETM) 1 is classically associated with mitochondria ionic regulation via pore formation across the mitochondrial membranes. LETM1 is highly expressed in many different types of tumours, especially in cancer stem cells, and is associated with poor prognosis^[144]^. As the C-terminal of LETM1 in the mitochondrial matrix has a 14-3-3-like motif, can this motif stabilize mitochondrial AANAT ^[145]^?

As PTEN-induced kinase (PINK) 1 phosphorylates and optimizes LETM1 function, the loss of PINK1 in GBM/GSC^[146]^ will modulate not only mitochondrial ionic regulation but potentially impact upon the mitochondrial melatonergic pathway. This requires investigation as potentially this links previously diverse bodies of data pertaining to GBM/GSC survival and chemoresistance, especially as Ca^2+^ dysregulation is evident in GBM mitochondria^[147]^.

Given Bmal1 and SIRT1 mutually induce each other in non-neoplastic cells^[129]^, it requires investigation as to the interactions of Bmal1 and sirtiuins in the regulation of mitochondria and the mitochondrial melatonergic pathway. Resveratrol, a SIRT1 inducer, affords protection in GBM/GSC and has recently been proposed as an adjunctive in GBM/GSC treatment^[148]^, with the induction of SIRT1 having effects that include regulation of the GBM/GSC microenvironment^[149]^. As noted above, resveratrol also inhibits AhR, suggesting that it will have an impact on the NAS/melatonin ratio. The interactions of sirtuins with resveratrol and the melatonergic pathway will be important to determine.

## Treatment implications

It is highly likely that melatonin per se, as well as its adjunctive use with chemotherapy, will have positive benefits on GBM/GSC patient outcomes^[150]^.

Ultrasound-induced local BBB disruption may allow for an increased availability of the wide array of factors that have been shown in preclinical studies to suppress GBM/GSC^[151]^, and may allow for an increased invasion of mast cells as well as other melatonin producing cells into the GBM/GSC microenvironment. Whether mast cells or other cells chemoattracted to the GBM/GSC microenvironment can be primed for an increase in melatonin release and/or for the inhibition of the ‘backward’ conversion of melatonin to NAS will be interesting to investigate.

Melatonin increases miR-149^[152]^, which is epigenetically suppressed in GBM^[153]^, with miR-149 and miR-128 increasing chemosensitivity to temozolomide^[154]^.

Given the efficacy of butyrate in the induction of senescence in GBM^[25]^ and apoptosis in GSC^[24]^ as well as increasing chemosensitivity and suppressing YY1^[31]^, it requires investigation as to the clinical utility of sodium butyrate in the management of GBM/GSC, including as an adjunctive to chemo-and radio-therapy.

Given that the maximal toxicity of temozolomide is optimized by the upregulation of the circadian gene, *Bmal1*^[5]^, it would seem likely that research to clarify the roles of circadian genes, circadian rhythm and the melatonergic pathway, will provide ways of improving clinical utility and treatment outcomes.

## Conclusion

Chemoresistance is common in GBM/GSC, being reflective of the plasticity of response and adaptation that is inherent in the tumour microenvironment. The above would indicate that the mitochondrial melatonergic pathway might be an important aspect, if not driver, of the plasticity in the intercellular interactions of the cells of the tumour microenvironment. The incorporation of the mitochondrial melatonergic pathway provides a conceptual frame of reference that better integrates the diverse array of previously disparate data on the biological underpinnings of GBM/GSC. This has implications for understanding the ubiquitous radio- and chemo-resistance that are evident in GBM/GSC as well as providing novel, and readily achievable, treatment targets.

## Declarations

### Availability of data and materials

Review of current literature.

### Copyright

© The Author(s) 2020.
